# Clinical presentation and proteomic signature of patients with *TANGO2* mutations

**DOI:** 10.1002/jimd.12156

**Published:** 2019-08-13

**Authors:** Nadja Mingirulli, Angela Pyle, Denisa Hathazi, Charlotte L. Alston, Nicolai Kohlschmidt, Gina O'Grady, Leigh Waddell, Frances Evesson, Sandra B. T. Cooper, Christian Turner, Jennifer Duff, Ana Topf, Delia Yubero, Cristina Jou, Andrés Nascimento, Carlos Ortez, Angels García‐Cazorla, Claudia Gross, Maria O'Callaghan, Saikat Santra, Maryanne A. Preece, Michael Champion, Sergei Korenev, Efsthatia Chronopoulou, Majumdar Anirban, Germaine Pierre, Daniel McArthur, Kyle Thompson, Placido Navas, Antonia Ribes, Frederic Tort, Agatha Schlüter, Aurora Pujol, Raquel Montero, Georgia Sarquella, Hanns Lochmüller, Cecilia Jiménez‐Mallebrera, Robert W. Taylor, Rafael Artuch, Janbernd Kirschner, Sarah C. Grünert, Andreas Roos, Rita Horvath

**Affiliations:** ^1^ Department of Neuropediatrics and Muscle Disorders Medical Center – University of Freiburg, Faculty of Medicine Breisgau Germany; ^2^ Department of General Pediatrics Adolescent Medicine and Neonatology, Medical Center – University of Freiburg, Faculty of Medicine Breisgau Germany; ^3^ Wellcome Centre for Mitochondrial Research Institute of Genetic Medicine, Newcastle University Newcastle upon Tyne UK; ^4^ Biomedical Research Department Leibniz‐Institut für Analytische Wissenschaften – ISAS – e.V Dortmund Germany; ^5^ Wellcome Centre for Mitochondrial Research Institute of Neuroscience, Newcastle University Newcastle upon Tyne UK; ^6^ Institute of Clinical Genetics and Tumor Genetics Bonn Germany; ^7^ Kid's Neuroscience Centre, Children's Hospital at Westmead Sydney New South Wales Australia; ^8^ Discipline of Child and Adolescent Health The University of Sydney Sydney New South Wales Australia; ^9^ Cardiology The Children's Hospital at Westmead Sydney New South Wales Australia; ^10^ John Walton Muscular Dystrophy Research Centre Institute of Genetic Medicine, Newcastle University Newcastle upon Tyne UK; ^11^ Department of Clinical Biochemistry, Genetics, Pediatric Neurology and Cardiology and Biobank Institut de Recerca Sant Joan de Déu and CIBERER, Instituto de Salud Carlos III Barcelona Barcelona Spain; ^12^ Birmingham Women's and Children's NHS Foundation Trust Birmingham UK; ^13^ Department of Inherited Disease St Thomas Hospital London UK; ^14^ South West Regional Metabolic Department Bristol Royal Hospital for Children Bristol UK; ^15^ Center for Mendelian Genomics and Program in Medical and Population Genetics Broad Institute of MIT and Harvard Cambridge Massachusetts; ^16^ Analytic and Translational Genetics Unit Massachusetts General Hospital Boston Massachusetts; ^17^ Centro Andaluz de Biología del Desarrollo Uníversidad Pablo de Olavide‐CSIC‐JA and CIBERER, Instituto de Salud Carlos III Madrid Spain; ^18^ Secció d'Errors Congènits del Metabolisme – IBC Servei de Bioquímica I Genètìca Molecular, Hospital Clínìc, IDIBAPS, CIBERER Barcelona Spain; ^19^ Neurometabolic Diseases Laboratory, Institut d'Investìgacío Biomedíca de Bellvitge (IDIBELL), and Centre for Biomedical Research on Rare Diseases (CIBERER), Instituto de Salud Carlos III Madrid Spain; ^20^ Catalan Institution of Research and Advanced Studies (ICREA) Barcelona Spain; ^21^ Children's Hospital of Eastern Ontario Research Institute, University of Ottawa Ottawa Ontario Canada; ^22^ Division of Neurology, Department of Medicine The Ottawa Hospital Ottawa Ontario Canada; ^23^ Pediatric Neurology University Children's Hospital, University of Duisburg‐Essen, Faculty of Medicine Essen Germany; ^24^ Department of Clinical Neurosciences University of Cambridge Cambridge UK

**Keywords:** fatty acid metabolism, metabolic encephalomyopathy, mitochondrial dysfunction, proteomic analysis, rhabdomyolysis, TANGO2

## Abstract

Transport And Golgi Organization protein 2 (TANGO2) deficiency has recently been identified as a rare metabolic disorder with a distinct clinical and biochemical phenotype of recurrent metabolic crises, hypoglycemia, lactic acidosis, rhabdomyolysis, arrhythmias, and encephalopathy with cognitive decline. We report nine subjects from seven independent families, and we studied muscle histology, respiratory chain enzyme activities in skeletal muscle and proteomic signature of fibroblasts. All nine subjects carried autosomal recessive *TANGO2* mutations. Two carried the reported deletion of exons 3 to 9, one homozygous, one heterozygous with a 22q11.21 microdeletion inherited *in trans*. The other subjects carried three novel homozygous (c.262C>T/p.Arg88*; c.220A>C/p.Thr74Pro; c.380+1G>A), and two further novel heterozygous (c.6_9del/p.Phe6del); c.11‐13delTCT/p.Phe5del mutations. Immunoblot analysis detected a significant decrease of TANGO2 protein. Muscle histology showed mild variation of fiber diameter, no ragged‐red/cytochrome c oxidase‐negative fibers and a defect of multiple respiratory chain enzymes and coenzyme Q_10_ (CoQ_10_) in two cases, suggesting a possible secondary defect of oxidative phosphorylation. Proteomic analysis in fibroblasts revealed significant changes in components of the mitochondrial fatty acid oxidation, plasma membrane, endoplasmic reticulum‐Golgi network and secretory pathways. Clinical presentation of *TANGO2* mutations is homogeneous and clinically recognizable. The hemizygous mutations in two patients suggest that some mutations leading to allele loss are difficult to detect. A combined defect of the respiratory chain enzymes and CoQ_10_ with altered levels of several membrane proteins provides molecular insights into the underlying pathophysiology and may guide rational new therapeutic interventions.

## 1 BACKGROUND

The family of TANGO proteins plays a crucial role in the interaction of the Golgi apparatus and the endoplasmic reticulum (ER). Bi‐allelic *TANGO2 (NM_152906.5)* mutations have been identified as a cause of a pediatric disease with multi‐organ involvement.^1^, ^2^ *TANGO2* was first described in a genome‐wide RNA‐mediated interference screen in *Drosophila*.^3^ TANGO2 is localized to the Golgi and the cytoplasm. Its depletion leads to the fusion of Golgi membranes with the ER.^4^ Consequently, increased ER stress was postulated to be a contributing factor to the disease mechanism of TANGO2 deficiency.^1^ A total of 29 cases have been reported to date (Tables S1 and S2). The most frequent mutation in patients with European ancestry (minor allele frequency [MAF] 0.0013) is a 34 kB deletion of exons 3 to 9, present in 17 patients. This change was present in one additional patient who also carried a de novo 22q11.21 deletion inherited in *trans*. A missense mutation (c.460G>A/p.Gly154Arg) has a 0.0026 MAF in the Hispanic/Latino population and was present in six of the reported patients.^1^, ^5^ Other mutations are rare and uniquely identified within single families. Independent of the underlying mutation, all reported cases show a remarkably similar neurological phenotype in combination with severe recurrent metabolic crises resulting in rhabdomyolysis, severe encephalopathy and life‐threatening arrhythmias. The age of onset varied from 4 months to 8 years and in most cases neurological symptoms were present before the first critical metabolic derangement. Biochemical findings during metabolic crisis included lactic acidosis, hypoglycemia, ketonuria—similar to mitochondrial diseases. However, the presence of rhabdomyolysis with very high creatine kinase (CK) levels with non‐specific increase of acylcarnitines and dicarboxylic acids were hallmarks of TANGO2 deficiency.^1^, ^5^

To date, very little is known about the function of the TANGO2 protein and its role in metabolic decompensation. However, the clinical presentation and metabolic changes raised the possibility that mitochondrial impairment and an increase of ER stress may be underlying pathophysiological factors.^4^, ^5^ Notably, both, the human and the mouse orthologue of *TANGO2* (NM_152906.6), T10 (NM_138583.2)^3^, ^6^ have a mitochondrial targeting sequence suggesting mitochondrial localization.

To further elucidate the clinical presentation and pathophysiological background of the disease, we report nine new patients with studies on the proteomic signature of TANGO2‐deficient fibroblasts.

## 2 CASE REPORTS

We report nine novel TANGO2 cases—four male and five female patients from seven different families, summarized in Table 1. All patients presented with a very similar clinical phenotype of progressive neurodevelopmental decline and recurrent metabolic crisis, combined with distinct biochemical findings. Five out of seven patients showed no complications during pregnancy, delivery or neonatal period, although two individuals (subject 1 and subject 3.2) were small for gestational age and presented with severe microcephaly at birth (head circumference 31 cm, sodium dodecyl sulfate (SDS) ‐2, 9, and 32 cm, SDS ‐2,8 respectively). Expanded newborn screening for inherited metabolic diseases was normal in four out of seven cases. The first symptoms occurred before 2 years of age when psychomotor retardation became evident in all patients, commonly associated with mild generalized muscular hypotonia, difficulties or inability to ambulate and cognitive impairment with poor or absent speech development.

**Table 1:** Clinical and diagnostic features of subjects in this study

Clinical features	Subject 1 Family 1	Subject 2.1 Family 2	Subject 2.2 Family 2	Subject 3.1 Family 3	Subject 3.2 Family 3	Subject 4 Family 4	Subject 5 Family 5	Subject 6 Family 6	Subject 7 Family 7
Sex	Female	Female	Female	Male	Male	Male	Female	Female	Male
Consanguineous	Yes	No	No	Yes	Yes	Yes	No	Yes	No
Ethnicity	Caucasian	Hispanic	Hispanic	Caucasian	Caucasian	Arab	Caucasian	Afghan	Caucasian
Age of onset	6 mo	1 y	2 y	2 y	1 y	2 y	<1 y	6 mo	13 mo
Developmental delay at onset	Severe	Severe	Mild	Moderate	Moderate	Moderate	Moderate	Mild/moderate	Mild
Recurrent metabolic crises (age at first event)	Yes (6 mo)	Yes (5 y)	Yes (9 y)	Yes (2 y)	No	Yes (2 y)	Yes (5 y)	Yes (6 mo) normal at age 2 y	Yes (13 mo)
ECG changes/cardiac arrhythmias (long QTc/Ventr. tachycardia/Torsade de Pointe)	Yes	Yes	Yes	Yes	Yes	Yes	Yes	Yes	Yes initially, but repeat ECG and echo normal
Seizures	Yes	Yes	Yes	Yes	Yes	Yes	No	Yes	No
Movement disorder (ataxia/EPS)	Yes	Yes	Yes	Yes	Yes	Yes	No	Mild hypertonia in all four limbs	Lower limb stiffness
Structural brain abnormalities (global brain atrophy)	Yes	No	No	No	Microcephalic	Yes	Not reported	Not reported	No
Premature death	No	Yes	Yes	No	Yes	Yes	No	No	No
Lactate (mmol/L) (normal <2 mmol/L)	19.9	4.4	3.8	2.1	2,5	2.6	Yes	8	No
Hypoglycemia (mmol/L)	1.11	No	No	No	No	No	No	0.9	No
Ketonuria	Yes	Yes	Yes	Yes	Yes	Yes	Yes	Yes	Not reported
Acylcarnitine elevation	C2↑ C10↑ C14**↑** C14:1↑ C14:2↑ C16:1↑ C18:1↑	Normal	Normal	Normal	Normal	C3↑ mild	C14:1 **↑,** C12‐C18 **↑**	C14**↑,** C14:1 at upper limit of reference range	Normal
Ammonia (μmol/L) (normal 11‐32)	829	121	120	163	Not reported	No	55	No	Not reported
ALT (U/L) (normal 7‐56)	12	1931	1536	2810	40	4268	1273	427	~3000
AST (U/L) (normal 10‐40)	95	3512	3032	2284	40	7326	3211	Not reported	~7000
Max. CK ↑ (U/l) (normal 22‐198)	10 166	261 716	200 000	221 000	419	260 000	>100 000	82 973	>400 000
TSH (μU/mL) (normal 0.4‐4)	6.5	3.7	15.2	17.3	28,6	11.2	33.8	48	Not reported
*TANGO2* mutations	Hom. c.262C>T, p.Arg88*	Hemizygous c.11_13delTCT, p.Phe5del		Hom. c.380+1G>A		Hom. c.220A>C,p.Thr74Pro	Hemizygous exon 3–9 deletion	Hom. c.6_9del, p.Phe6del	Hom. exon 3–9 deletion

Abbreviations: ALT, alanine aminotransferase; AST, aspartate transaminase; CK, creatine kinase; ECG, echocardiogram; EPS, extrapyramidal motor signs; Hom, homozygous; m, months; TSH, thyroid‐stimulating hormone; y, years.

All patients had metabolic encephalomyopathic crises between 6 months (subject 1) and 9 years (subject 2.2) of age. In the intervals the patients achieved a stable metabolic status with nearly normalized laboratory values and a good general condition. However, a progressive neurological decline with severe global psychomotor retardation was present in most patients except for subject 2.2, who only had moderate intellectual disability with learning difficulties, requiring special education. Most reported subjects developed epilepsy and progressive ataxia with dysarthric speech, inability to walk and spasticity. Brain MRI was abnormal, showing a mild global brain atrophy in two patients (subjects 1 and 4) and microcephaly in subject 3.2 (Figure S1).

The metabolic crises were characterized by an acute onset, often triggered by infection or physical stress. Serum biochemistry testing showed a very similar pattern in all cases with very high (419‐400 000 U/L) CK levels, lactic acidosis, elevated transaminases and hyperammonemia. Hypoglycemia was documented in two patients (subject 1 and subject 6) and non‐specific elevation of acylcarnitines and dicarboxylic acids in four patients (subject 1, 4, 5, and 6) suggestive of generalized energy deficiency. Ketonuria and thyroid stimulating hormone (TSH) elevation was present in 8/9 cases. TSH elevation was treated with L‐Thyroxin in subject 2.1, 2.2, 3.1, 3.2, 4, and 6. Cerebrospinal fluid analysis did not reveal signs of metabolic derangement in any patients.

In line with the characteristic biochemical alterations during metabolic crises, all patients showed overlapping clinical presentations with repeated bouts of massive rhabdomyolysis, hepatopathy and often severe encephalopathy with unresponsiveness and generalized seizures, requiring intensive care unit treatment. Some of our reported patients developed life‐threatening arrhythmias (Torsade de Pointes) on the basis of a Long‐QT‐Syndrome. Malignant arrhythmias led to premature death in three cases: subject 2.2 at the age of 12 years after she had experienced several encephalomyopathic episodes, subject 3.2 at the age of 5 years without any prior metabolic derangements and despite propranolol therapy and subject 4 at the age of 8 years. Subject 2.1 survived various arrhythmic episodes, developed left ventricular failure and died because of a bacterial superinfection resulting in multi‐organ failure at the age of 7 years. Subject 3.1, regardless of continuous propranolol therapy, developed repetitive life threatening arrhythmias which he survived. Subjects 1, 4, 5, and 7 showed a long QT syndrome in ECG, and subject 4 died at the age of 8 years due to cardiac complications. Hypertrophic cardiomyopathy was observed in subject 5.

Autopsy of subject 2.1 and 2.2 revealed diffuse edema of the central nervous system, myocardial softening, with dilatation of cavities. The histological examination showed mild disarray of overall architecture of myocytes with hypertrophy and mild interstitial fibrosis. In skeletal muscle, necrosis was present, in line with repeated episodes of rhabdomyolysis. In liver, hepatocyte cytoplasm appeared granular with few fat vacuoles in subject 2.1 without abnormal glycogen storage (Figure S2).

The clinical phenotype and unique biochemical profile were highly suggestive of a metabolic muscle disease; therefore, muscle biopsy has been performed in seven cases from six different families (subject 1, 2.1, 2.2., 3.1, 4, 5, 7). Following the working diagnosis of an underlying metabolic myopathy (potentially mitochondrial), anabolizing management was performed during the acute crises leading to a slow stabilization of the metabolic derangement in the majority of episodes. The reported patients received supplemental therapy consisting of riboflavin (vitamin B_2_), carnitine and thiamine (vitamin B_1_) (subject 1), carnitine, vitamin A/E/B2 (subject 2.1, 2.2), or vitamin B1 and carnitine respectively (subject 3.1 and 3.2). This supplemental therapy was continued in the interval but could not prevent repetitive metabolic decompensations in subject 1, 2.1, 2.2, and 3.1. The pharmaceutical regimen was expanded with Q_10_ substitution for subject 1 and subject 3.1, with regard to reduced CoQ_10_ levels, which helped prevent further metabolic derangements to date. Subject 4 did not receive any specific therapy.

## 3 METHODS

### 3.1 Genetic studies

Whole exome sequencing (WES) was applied in subjects 1, 3.1, 3.2, and 4. Whole genome sequencing (WGS) was performed in subjects 2.1 and 2.2 (see Supplementary Materials). In subjects 5, 6, and 7, targeted sequencing of the *TANGO2* gene (NM_152906.5) was undertaken using BigDye Terminator v3.1 Sanger sequencing on an ABI3130xl.

### 3.2 Muscle histology and biochemistry

Muscle histology and spectrophotometric analysis of the respiratory chain enzymes was performed as previously described.^7^, ^8^ Muscle CoQ_10_ analysis was done by high performance liquid chromatography with electrochemical detection, as reported^9^

### 3.3 Cell culture

Primary human fibroblasts from patients and controls were cultured in minimum essential media supplemented with 10% fetal bovine serum, 1% penicillin/streptomycin, 2 mM L‐glutamine, 1x non‐essential amino acids, 1x minimum essential medium vitamins, 1 mM sodium pyruvate, 50 μg/mL uridine (ThermoFisher Scientific), at 37°C, in a humidified 5% CO_2_ atmosphere.

### 3.4 Immunoblotting

Immunoblotting was performed as described previously.^10^ Primary antibodies were Anti‐C22orf25 antibody (Abcam, ab87576), GAPDH antibody (FL‐335) (Santa Cruz, sc‐25778), alpha tubulin (Abcam, ab7291), Anti‐VDAC1 (Abcam, ab14734). Oxidative phosphorylation (OXPHOS) proteins were probed using Total OXPHOS Human WB Antibody Cocktail (Abcam, ab110411) in subjects 1, 2.1 2.2, 4, 6. In Family 2 proteins were detected fluorescently using the Total OXPHOS Antibody Cocktail and Anti‐VDAC1/Porin antibody (Abcam, ab154856) using fluorescently labeled secondary antibodies (Licor IRDye 800CW anti‐rabbit, 926‐32211 and Licor IRDYE 680RD anti‐mouse, 925‐68070) and visualized on a Licor Odyssey CLx.

### 3.5 Golgi trafficking

Golgi‐apparatus (GA)‐ER retrograde transport was analyzed by immunofluorescence using anti‐GM130 antibody followed by microscopy in fibroblasts of subject 2.1 and 2.2 as well as control cells. To monitor GA‐ER retrograde vesicle transport the number of cells with assembled GA was counted at different time points upon Brefeldin A (BFA) treatment, an inhibitor of the ER‐Golgi anterograde transport.^11^ For each experimental condition 100 to 125 cells were counted. The results were expressed as percentage of cells with assembled GA.

### 3.6 Proteomic analysis

In total six samples (three TANGO2‐patient derived fibroblasts [subjects 1, 3.1, and 4] and three control fibroblast lines) were processed independently. Sample preparation was performed using filter‐aided sample preparation as described previously.^12^ Data analysis of the acquired label free quantitative MS was performed using the Progenesis LC‐MS software from Nonlinear Dynamics (Newcastle upon Tyne, UK) (see Figure 2 and supplementary material).

## 4 RESULTS

### 4.1 Genetic studies

In subject 1 mutation analysis of several candidate genes was negative including *POLG* and the known CoQ_10_ biosynthesis genes. WES identified a homozygous nonsense mutation (c.262C>T, p.Arg88*) in the *TANGO2 (NM_152906.5)* gene (Figure S3). This variant is predicted to result in a premature stop codon and not present in ExAc or Bravo databases. In subject 2.1 and 2.2 focused single gene analysis comprised NGS panel for genes associated with channelopathies, Long QT syndrome, *DMD, LPIN1,* and *RYR1* were negative. WGS revealed a heterozygous *TANGO2* mutation (c.11_13delTCT, p.Phe5del) and a putative gene deletion causing a loss in the second allele, which could not be detected by analysis of structural variation. Subject 3 and 3.1. are homozygous carriers of the mutation c.380+1G>A, affecting the exon 5 essential splice site (Figure S3). In subject 4 targeted sequencing of *DMD* and *LPIN1* were normal and no mitochondrial deletions were detected. After the first *TANGO2* mutations had been published, WES results were re‐evaluated, detecting a novel homozygous missense mutation in *TANGO2*: c.220A>C, p.Thr74Pro (Figure S3). In subject 5, no pathogenic mutations were detected in *ACADVL*, *ACAD9* or *LPIN1*. WES initially revealed no clear cause for these symptoms. Targeted sequencing of the *TANGO2* gene revealed a hemizygous deletion of exons 3 to 9, proven to be inherited from her father. The second mutation, which resulted in allele loss could not be identified. Subject 6 underwent targeted Sanger sequencing of the coding regions of *TANGO2* revealing a homozygous in‐frame deletion c.6_9del, p.Phe6del. In Subject 7, a heterozygous mutation in *VLCAD* was detected but not considered relevant as VLCAD enzyme activity was normal in the cultured fibroblasts. Targeted sequencing of the *TANGO2* gene revealed a homozygous deletion of exons 3 to 9.

### 4.2 Muscle histology and biochemistry

Muscle histology and histochemistry of subjects 1, 2.1, 2.2, 3.1, and 4 revealed a mild variation of fiber diameter but no ragged‐red fibers or COX‐deficient fibers. All samples showed a variable extent of regenerative and degenerative changes including necrosis, consistent with the history of rhabdomyolysis. Muscle histology for subject 1 shows mild variation of fiber diameters in the H&E staining (A) and no signs of mitochondrial accumulation in trichrome staining (B) (Figure S2). In subject 4, there was a significant type 2 fiber predominance, up to 80%. All samples were taken in a symptom‐free interval without acute rhabdomyolysis. In subject 1 electron microscopic examination was performed and did not show any relevant alterations of mitochondrial morphology. In subject 5, muscle biopsy analysis revealed significant lipid accumulation similar to defects of fatty acid oxidation, which is a non‐specific finding. No muscle biopsy was taken from subject 6. The muscle biopsy from subject 7 showed evidence of previous muscle fiber degeneration and necrosis, likely due to repeated episodes of muscle fiber breakdown with rhabdomyolysis. Electron microscopy results did reveal large areas of degenerative and regenerative fibers with clumping of mitochondria and some mitochondria appearing enlarged, with no inclusions observed and without typical changes of primary mitochondrial dysfunction.

**Figure 1:**
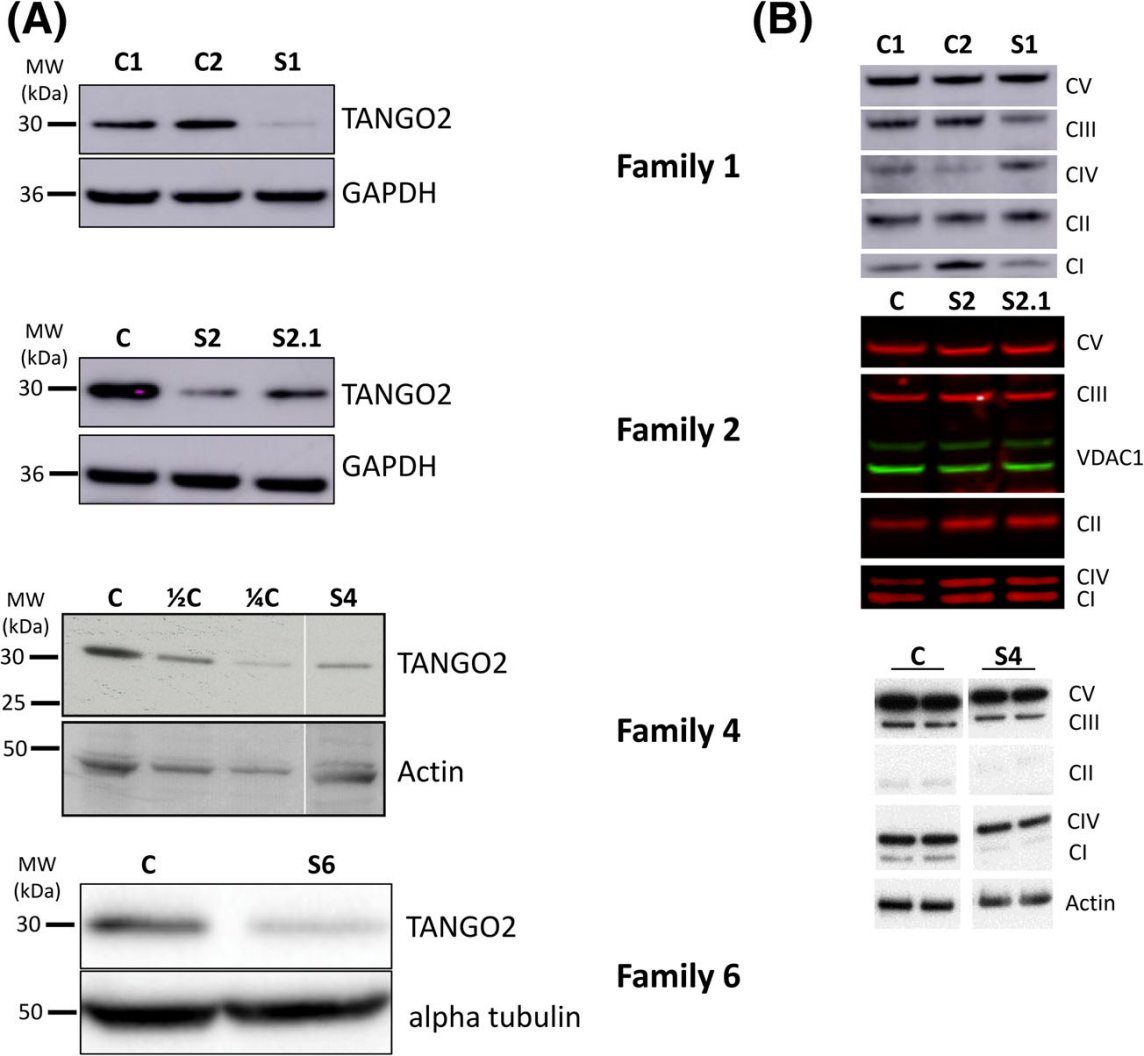
A, Immunoblotting for TANGO2 protein in control fibroblasts (C) and patient fibroblasts (P) in Families 1, 2, and 4. In Family 6, TANGO2 protein levels in muscle lysates of two controls and two patients. Western blotting was performed in triplicates. B, Western blot analysis of OXPHOS complex subunits performed in total protein lysates from primary fibroblasts in the patients in Families 1 and 2, and in muscle lysates from patients and controls in Family 4

OXPHOS activities were measured in several cases, although no uniform pattern of changes was identified across all patients (Table S3). In subject 1, biochemical assessment of the respiratory chain enzyme activities revealed a combined decrease involving complexes I and IV, with an associated CoQ_10_ deficiency (Table S3). A second muscle biopsy taken a few years later confirmed these earlier findings (results not shown). Subject 2 showed multiple deficiencies in mitochondrial respiratory chain enzyme activities in muscle, relative to an observed increased citrate synthase activity while investigations in fibroblasts were normal. CoQ_10_ in muscle was decreased. In subject 4 muscle respiratory chain activities were all normal, although western blot analysis of the OXPHOS complexes revealed a subtle reduction in important subunits of complexes II, III, IV (Figure 1B). In subject 3.1 and subject 3.2 respiratory chain activity was not analyzed. Respiratory chain enzyme activities in subjects 5 and 7 were normal.

### 4.3 Immunoblotting

In subject 1 TANGO2 protein levels in primary fibroblasts were markedly reduced when compared to two different controls, although we still detected a very slight residual band, which is unexpected in the case of a homozygous nonsense mutation (Figure 1). In subject 2.1 we found similar results with markedly reduced TANGO2 protein levels as well as in subject 4 and subject 6 with an underlying missense mutation (Figure 1A).

Western blot analysis of total protein lysate from fibroblasts of subject 1 showed a significant decrease in steady state levels of OXPHOS components of complex I (*P* = .034812) and complex III (*P* = .003068) when compared to two control fibroblast cell lines. Subject 4 presented a reduction in OXPHOS complexes II, III, IV (Figure 1B). Whereas western blot analysis from subject 2.1 and 2.2 showed a non‐specific increase of all OXPHOS complexes as a possible compensatory reaction. The defect of OXPHOS complexes was not linked to any specific *TANGO2* genotype.

### 4.4 Golgi trafficking

To determine whether the functionality of the GA‐ER retrograde vesicle transport could be affected in TANGO2 deficient cells we monitored the disassembly of the GA upon BFA treatment in subjects 2.1.and 2.2. Our results showed that 5 to 10 minutes after addition of BFA to the culture medium the GA was disassembled in the 55% of the control cells. In contrast, in TANGO2 patients the amount of cells with disassembled GA at the same time points was reduced to 33% (Figure 3). Although 60 minutes after BFA treatment all cells showed disassembled GA in both control and patient cells, the delayed disassembly detected at earlier time points is suggestive of a possible impairment in the retrograde GA‐ER trafficking in TANGO2 patient fibroblasts (Figure 3).

### 4.5 Proteomic signature of fibroblasts carrying autosomal recessive *TANGO2* mutations

**Figure 2:**
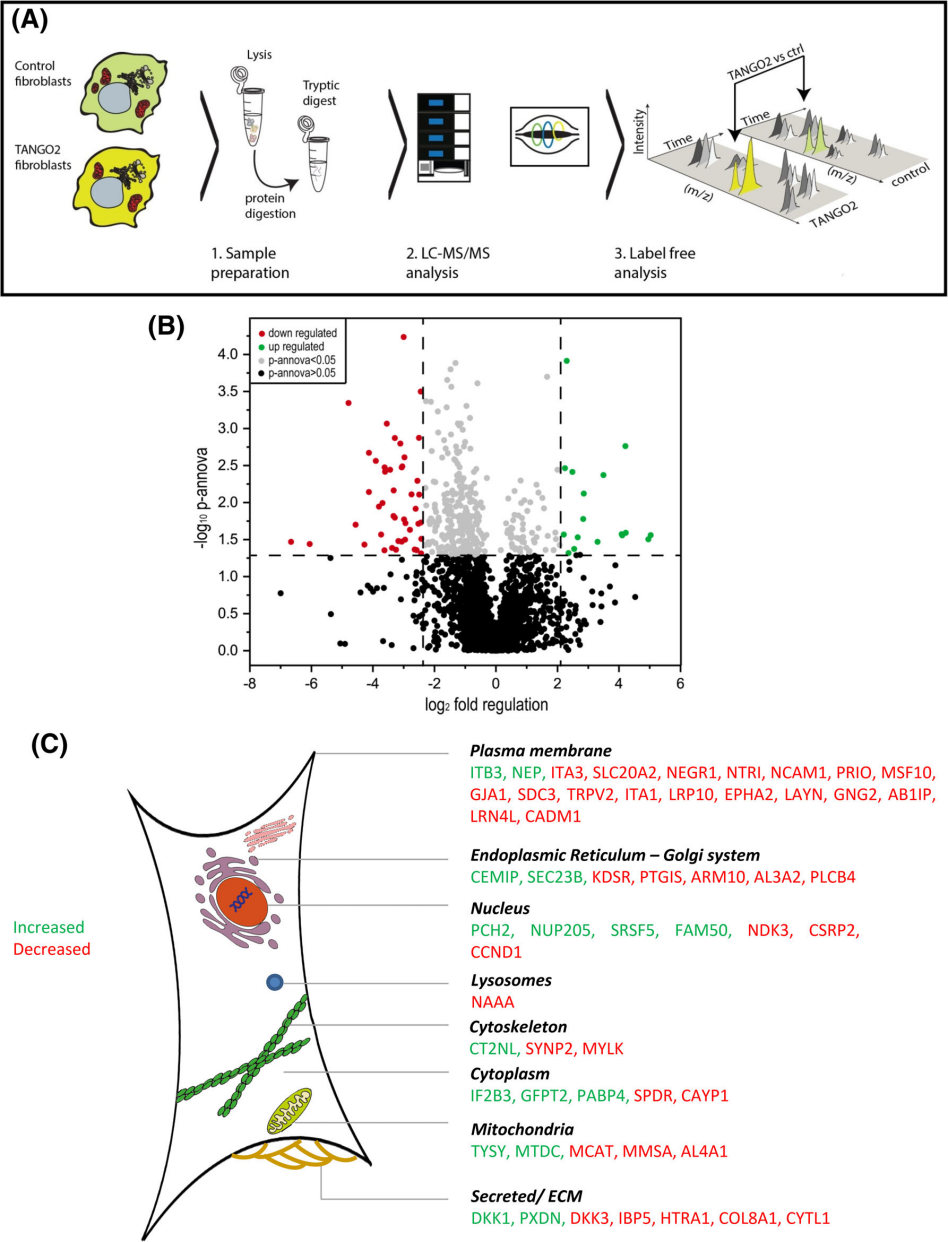
Proteomic profiling of patient derived fibroblasts: A, Proteomic workflow applied to identify the proteins affected by the TANGO2‐deficiency in fibroblasts. B, Volcano‐plot of proteomic findings. Red dots represent proteins showing a statistically significant decrease, whereas green dots represent proteins with a statistically significant increase in abundance. C, Subcellular distribution of affected proteins

Here, we analyzed the proteomic signature of our patients` fibroblasts (subjects 1, 3.1, and 4) using a label‐free approach (Figure 2.^13^ We found that 2.3% of the quantified proteins (55 out of 2355) were significantly differentially abundant upon *TANGO2* mutations in fibroblasts: 16 (29%) of these proteins were increased and 39 (71%) decreased. The affected proteins are localized in the ER‐Golgi network (eg, cell migration‐inducing and hyaluronan‐binding protein CEMIP, protein transport protein Sec23B), the secretory pathway (eg, peroxidasin homolog PXDN, serine protease HTRA1), the plasma membrane (eg, gap junction protein alpha‐1 CXA1, major prion protein PRIO, sodium dependent phosphate transporter 2 S20A2) and in mitochondria (eg, aldehyde dehydrogenase 4 AL4A1) (Figure 2). In addition, significant changes were detected in proteins involved in fatty acid oxidation (eg, mitochondrial carnitine/acylcarnitine carrier protein MCAT, Fatty aldehyde dehydrogenase AL3A2) and amino acid metabolism (eg, methylmalonate‐semialdehyde dehydrogenase MMSA). *SLC25A20,* encoding a mitochondrial carnitine/acylcarnitine carrier protein was decreased in TANGO2‐patient derived fibroblasts. For an overview on the regulated proteins and proposed functions, see Table S4. Interestingly, protein levels for OXPHOS complexes I, III, IV, and V were not significantly altered in fibroblasts, whereas complex II and TANGO2 protein were not detected by proteomic analysis in normal and mutant fibroblasts despite being detectable by Western blotting.

## 5 DISCUSSION

We report nine children presenting during early childhood with severe psychomotor developmental delay, refractory seizures and recurrent metabolic crises with lactic acidosis, accompanied by rhabdomyolysis, cardiac arrhythmias and encephalopathy. Next generation sequencing revealed five novel mutations, and we show that four mutations result in a relevant reduction or dysfunction of TANGO2 protein in fibroblasts, suggesting a loss of function disease mechanism.

Previous studies utilizing fibroblasts derived from patients with *TANGO2* mutations suggested ER stress and altered Golgi volume density and noted defective mitochondrial fatty acid oxidation.^1^, ^5^ In two of our cases biochemical investigation revealed a reduced activity of different OXPHOS complexes in skeletal muscle (subject 1, 2.1) and normal OXPHOS activity in subject 4 (Table S3). These findings are underscored by Western blot analysis of OXPHOS complex subunits showing significant reduction of complexes I and III in subject 1 and II, III, IV in subject 4, respectively (Figure 1). Moreover, respiratory chain analyses showed CoQ_10_ deficiency in subject 1 and 2.1, while in the other reported subjects CoQ_10_ levels were not assessed. This CoQ_10_ reduction might be a secondary effect of the fatty acid oxidation impairment, similar to CoQ_10_ deficiency in multiple acyl‐CoA dehydrogenase deficiency.^14^, ^15^ We initiated a CoQ_10_ and riboflavin supplementation in subjects 1 and 3.1 which resulted in clinical improvement and stabilization without any further metabolic crisis up until now, however assessing treatment with CoQ_10_ and measuring CoQ_10_ in additional patients is needed before our findings can be generalized.

To date a total of 29 patients have been reported with bi‐allelic *TANGO2* mutations prior to this publication, all of which show a strikingly similar clinical phenotype. However, the biochemical phenotype is more variable. Except for an isolated mild reduction of complex I in one case^1^ the majority of the reported cases showed essentially normal mitochondrial studies^2^ without clear respiratory chain dysfunction (15 out of 18 cases in which mitochondrial studies were performed, Table S1). We performed proteomic profiling in fibroblasts, which indicated altered fatty acid oxidation, folic acid and pyrimidine metabolism in mitochondria.^16^ Impaired fatty acid oxidation was suggested by decrease of fatty aldehyde dehydrogenases (ALDH3A2, ALDH4A1, ALDH6A1^17^;) and *SLC25A20,* encoding a mitochondrial carnitine/acylcarnitine carrier protein.^18^, ^19^ Mutations in these genes lead to severe neurological abnormalities, developmental delay, cardiomyopathy, arrhythmias, skeletal muscle damage, liver dysfunction, and episodes of life‐threatening coma,^20^, ^21^ many of these clinical findings are also present in TANGO2‐deficient patients.

**Figure 3:**
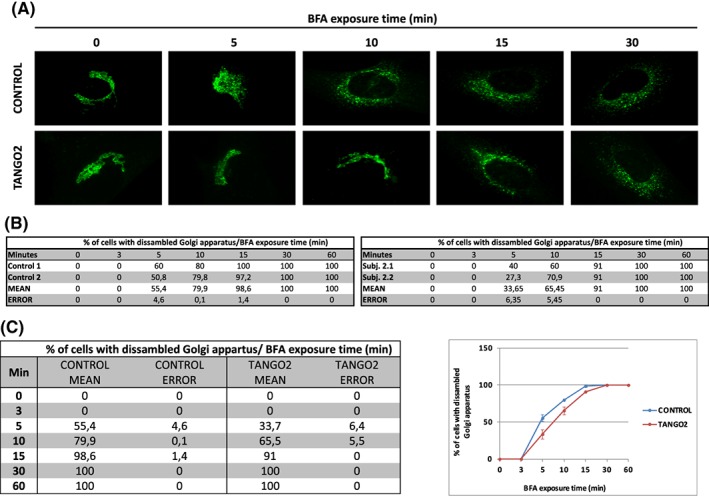
Golgi apparatus (GA)‐to‐endoplasmic reticulum (ER) retrograde membrane flow. A, Immunofluorescence using anti‐GM130 antibody followed by microscopy in TANGO2 patient fibroblasts (subject 2.2) and control cells. B, To monitor GA‐ER retrograde vesicle transport the number of assembled GA at different time points upon Brefeldin (BFA) treatment (in percent) in both subjects (subject 2.1 and 2.2) and controls. C, Mean number of assembled GA at different time points upon BFA treatment (in percent) and error. Five and 10 minutes after BFA addition to the culture medium, a reduction of around 20% in the amount of cells with assembled GA was observed in both patients compared with control cells

Our proteomics results underline the impaired function of the protein processing machinery (Figure 2, Table S4) as earlier described,^4^ and Golgi trafficking (Figure 3). In addition, the proteomic signature of *TANGO2*‐mutant fibroblasts suggests an effect of the perturbed ER‐Golgi system on target proteins (HTRA1, ITA1, ITA3, NCAM1, NEGR1, NTRI, PRIO, S20A2, and SYNP2). SYNP2 modulates F‐actin networks^22^ and may contribute to skeletal muscle vulnerability and rhabdomyolysis in TANGO2 deficient patients. SDC3 organizes cell shape through actin cytoskeleton and regulates adult myogenesis,^23^ MYLK acts as a calcium/calmodulin‐dependent myosin light chain kinase and CAVN2 plays an important role in caveolar biogenesis and morphology. Caveolinopathy, a muscle disease based on mutations in Caveolin‐3 can also present with muscle pain and rhabdomyolysis,^24^ clinical manifestations in TANGO2‐patients. The TANGO2‐related cardiac phenotype is suggested by decrease of CXA1/CX43/GJA1.^25^ Thus, our data indicate that mitochondrial and ER‐Golgi function along with proper processing of secreted and plasma membrane proteins and fatty acid oxidation are predominantly affected by loss of functional TANGO2, highlighting these processes as relevant for TANGO2 pathophysiology and most likely for TANGO2‐dependent disease manifestation.

Taken together, proteomic signature of TANGO2‐patient derived fibroblasts suggests new clues into the underlying pathophysiology providing a rational for future targeted therapy. Avoidance of typical precipitants (catabolic stress, especially during infection, trauma, vomiting) is recommended, although metabolic derangement did also occur in some patients *without* any identifiable trigger. Anabolizing management, treatment with vitamin B_1_, B_2_ and carnitine resulted in stabilization of metabolic crises in the reported patients, together with consequent treatment of organic complications (eg, hyperammonemia, seizures, cardiac arrhythmias). Two of the reported subjects additionally received CoQ_10_ and did not suffer any further metabolic derangements to date. This highlights that in addition to the impairment of ER‐Golgi, alterations of mitochondrial fatty acid oxidation, respiratory chain and amino acid pathways significantly contribute to the clinical presentations of TANGO2 deficiency.

## CONFLICT OF INTEREST

None of the authors has conflict of interests to declare.

## AUTHOR CONTRIBUTIONS

M.N. was involved in the collection of clinical data as well as interpretation of laboratory data and drafted the manuscript including Figures S1 and S2. P.A. performed the genetic and immunoblot analyses, drafted Figure S3 and S4. She also critically revised the manuscript. L.C.A., K.N., O.G., W.L., E.F., S.B.T.C., T.C., D.J., T.A., Y.D., J.C., N.A., O.C., A.G.‐C, G.C., O.M., S.S., M.A.P., C.M., K.S., C.E., P.G., M.D., T.K., N.P., R.A., T.F., S.A., P.A., M.R., S.G., L.H., C.J.‐M., R.W.T., A.R., J.K., and S.C.G.) were involved in data collection and interpretation and critically revised the manuscript. H.D. and R.A. performed and analyzed the proteomic analysis and drafted the corresponding part of the manuscript including Figures 2 and 3. H.R. was the main coordinator of the project, involved in planning and conduction of the study, supervised the experimental lab work and drafted the manuscript together with N.M.

## ETHICS STATEMENT

Informed consent was obtained from each participant approved by local ethical approvals and by the North Research Ethics (NRES) Committee Yorkshire & The Humber – Leeds Bradford 13/YH/0310. The samples are stored in the Newcastle Biobank of the MRC Centre for Neuromuscular Diseases. Consent for publication of these data was obtained from all patients/patient families. Documentation of approval from the Institutional Committee for Care and Use of Laboratory Animals (or comparable committee).

## Supporting information


**Figure S1.** Brain MRI in subject 1 at 6 months, 3 weeks after her first metabolic crisis had occurred (A, B) and 6 years (C) of age, during a symptom‐free interval. A, T2: Flair with multifocal diffusion restrictions accentuated in the left parieto‐occipital regions. B, T1: deficient myelinization of the adjacent u‐fibers. Widening of the inner and outer CSF spaces. C, T2: progressive widening of the inner and outer CSF spaces. Multifocal cystic lesions.

**Figure S2.** Muscle Biopsy and necropsy findings.
A, Muscle Biopsy in subject 1 at 6 months of age. (a) Hematoxylin & eosin (H&E) staining detected mild variation of fiber diameters; (b) COX activity was normal; c) trichrome staining was normal and did not detect signs of mitochondrial accumulation (no ragged red fibers). B, Autopsy findings at age 12 years: (a) H&E staining in heart muscle (subject 2.2) showed mild disarray of the overall architecture of the myocytes with hypertrophy and a mild interstitial fibrosis. (b) H&E stain in liver (subject 2.1) showed hepatocytes arranged in plates and the hepatocytes display polygonal morphology with well‐defined borders. Hepatocyte cytoplasm appears granular and clear with few fat vacuoles in subject 2.1 (black arrows). Absence of portal or porto‐portal fibrosis.

**Figure S3.** Pedigrees and Sanger sequencing electropherograms for index cases and parental samples, where available, for Families 1, 2, 3, and 6.

**Data S1.** Table S1. Reported mutations in individuals with bi‐allelic Transport And Golgi Organization protein 2 (TANGO2) mutations (1,2).
**Table S2.** Clinical and diagnostic features in the presented subjects.
**Table S3.** Respiratory chain activity in skeletal muscle.
