# Supplementary figures and images for: Clinical presentation and proteomic signature of patients with TANGO2 mutations
Source: J Inherit Metab Dis. 2019 Aug 13;43(2):297–308. doi: 10.1002/jimd.12156 (PMC7078914; doi:10.1002/jimd.12156)

## Slide 1
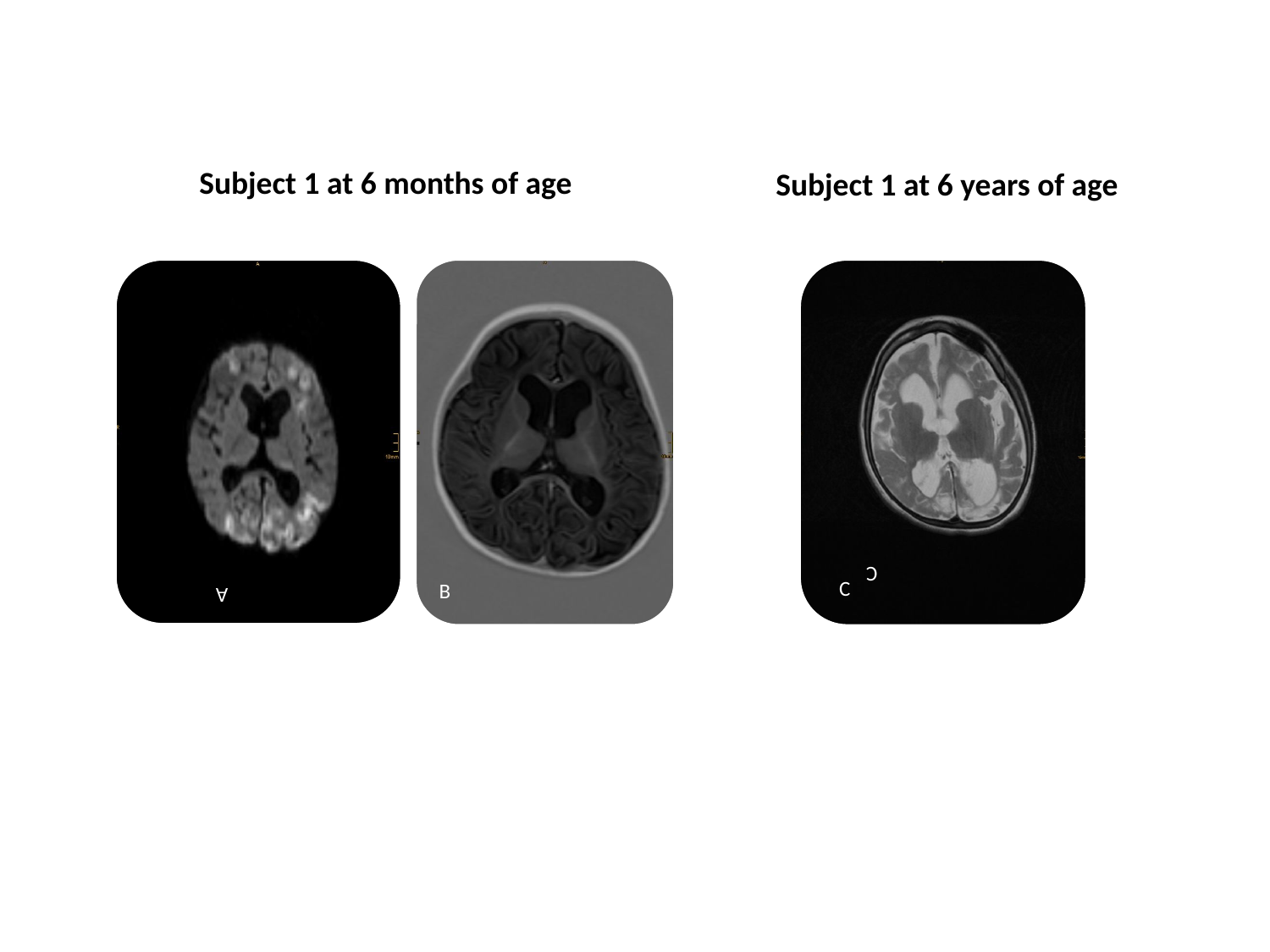

Subject 1 at 6 months of age
Subject 1 at 6 years of age
C

Supplement: Supplementary file 1 — Figure S1. Brain MRI in subject 1 at 6 months, 3 weeks after her first metabolic crisis had occurred (A, B) and 6 years (C) of age, during a symptom‐free interval. A, T2: Flair with multifocal diffusion restrictions accentuated in the left parieto‐occipital regions. B, T1: deficient myelinization of the adjacent u‐fibers. Widening of the inner and outer CSF spaces. C, T2: progressive widening of the inner and outer CSF spaces. Multifocal cystic lesions. [file JIMD-43-297-s001.pptx]

## Slide 1
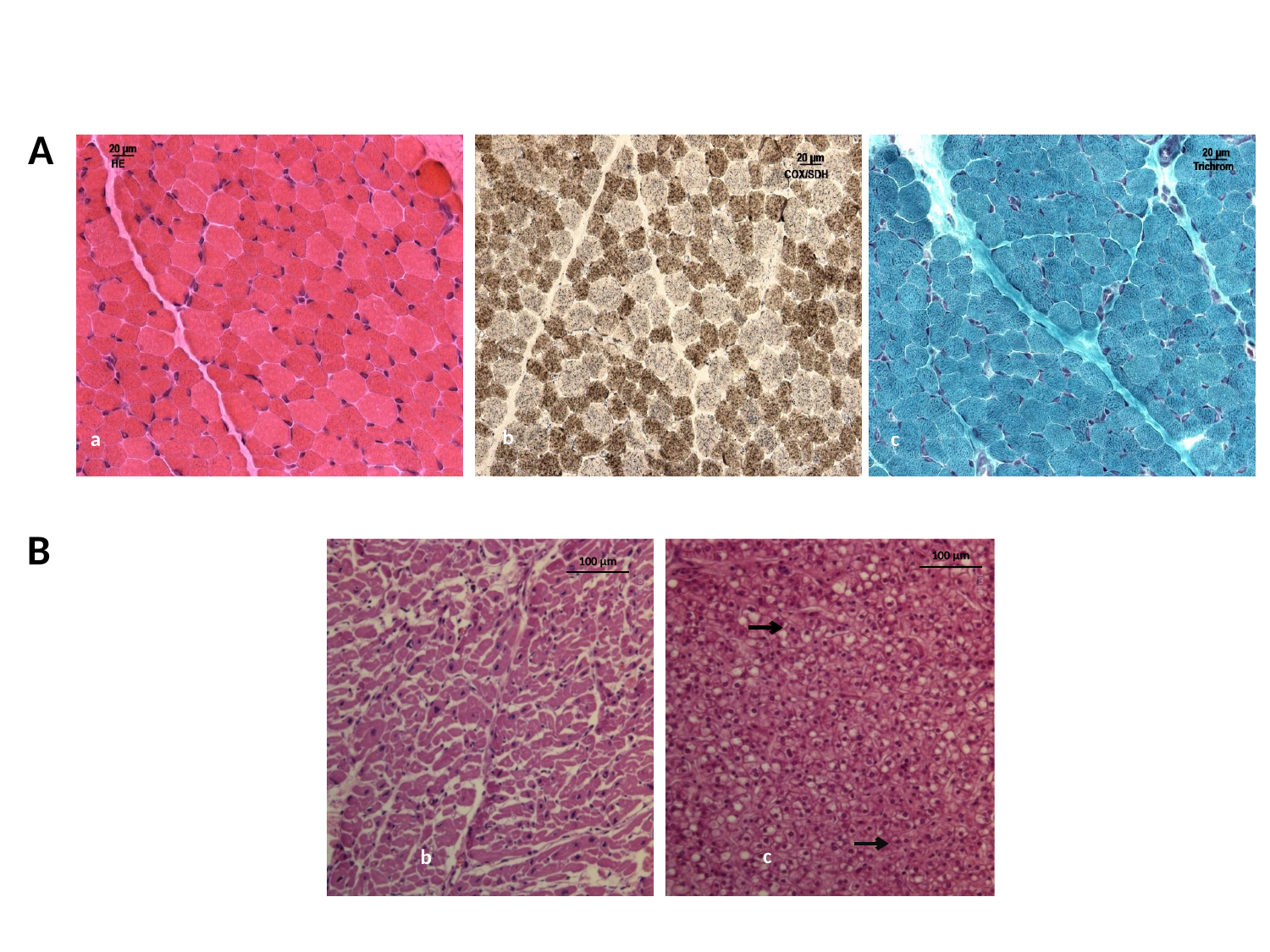

A
b
a
c
B
100 µm
a
E
100 µm
c
b
b
c
b

Supplement: Supplementary file 2 — Figure S2. Muscle Biopsy and necropsy findings. A, Muscle Biopsy in subject 1 at 6 months of age. (a) Hematoxylin & eosin (H&E) staining detected mild variation of fiber diameters; (b) COX activity was normal; c) trichrome staining was normal and did not detect signs of mitochondrial accumulation (no ragged red fibers). B, Autopsy findings at age 12 years: (a) H&E staining in heart muscle (subject 2.2) showed mild disarray of the overall architecture of the myocytes with hypertrophy and a mild interstitial fibrosis. (b) H&E stain in liver (subject 2.1) showed hepatocytes arranged in plates and the hepatocytes display polygonal morphology with well‐defined borders. Hepatocyte cytoplasm appears granular and clear with few fat vacuoles in subject 2.1 (black arrows). Absence of portal or porto‐portal fibrosis. [file JIMD-43-297-s003.pptx]

## Slide 1
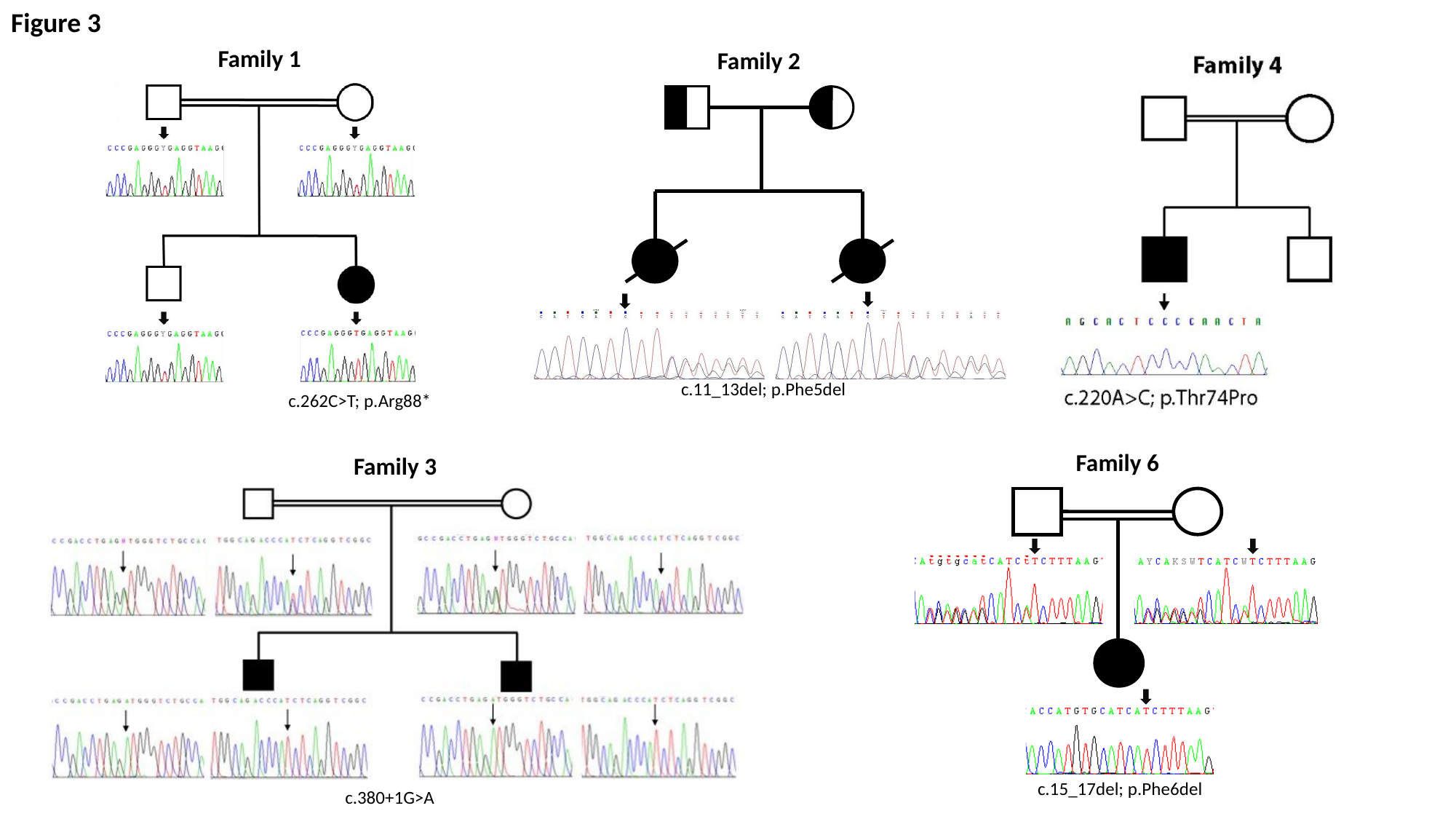

Figure 3
Family 1
c.262C>T; p.Arg88*
Family 2
c.11_13del; p.Phe5del
Family 6
c.15_17del; p.Phe6del
Family 3
c.380+1G>A

Supplement: Supplementary file 3 — Figure S3. Pedigrees and Sanger sequencing electropherograms for index cases and parental samples, where available, for Families 1, 2, 3, and 6. [file JIMD-43-297-s004.pptx]
