# Supplementary material for: Clinical presentation and proteomic signature of patients with TANGO2 mutations
Source: J Inherit Metab Dis. 2019 Aug 13;43(2):297–308. doi: 10.1002/jimd.12156 (PMC7078914; doi:10.1002/jimd.12156)
Supplement: Supplementary file 4 — Data S1. Table S1. Reported mutations in individuals with bi‐allelic Transport And Golgi Organization protein 2 (TANGO2) mutations (1,2). Table S2. Clinical and diagnostic features in the presented subjects. Table S3. Respiratory chain activity in skeletal muscle. [file JIMD-43-297-s002.docx]

**Supplemental Materials**

**Supplementary Methods**

### Genetic studies

WES was performed in genomic DNA using Illumina’s Truseq Rapid Exome Capture performed on a NextSeq500. Exome data were analyzed using previously described methodologies and bioinformatic filtering pipelines (Bartsakoulia et al, 2018). The pathogenicity of novel mutations was predicted by using the criteria established by the American College of Medical Genetics (<https://www.nature.com/gim/articles?type=acmg-practice-guidelines>). The Exome Aggregation Consortium (ExAC) Cohort was used as population reference data (<http://exac.broadinstitute.org>). WGS was performed as follows, DNA libraries were prepared with the KAPA Library Preparation kit (Kapa Biosystems). Sequencing was performed on an Illumina HiSeq2000 sequencer (Illumina Inc.) in paired-end mode, 2x126bp, according to standard Illumina operation procedures in the Centro Nacional de Análisis Genómico in Barcelona (CNAG). Sequences were aligned to hg19 by Burrows-Wheeler Aligner (BWA mem) and single variants and insertions/deletions (Indels) were identified using GATK, applying GATK’s best practices for germline SNP & Indel discovery in WES and annotated by Annovar software.

### Proteomics studies

### Following materials were purchased from Sigma-Aldrich, Steinheim, Germany: ammonium hydrogen carbonate (NH4HCO3), guanidine hydrochloride (GuHCl), iodoacetamide (IAA), and urea. Tris base was obtained from Applichem Biochemica, Darmstadt, Germany. Sodium dodecyl sulfate (SDS) was purchased from Carl Roth, Karlsruhe, Germany. Dithiothreitol (DTT), EDTA-free protease inhibitor (Complete Mini) tablets were bought from Roche Diagnostics, Mannheim, Germany. Sodium chloride (NaCl) and calcium chloride (CaCl2) were from Merck, Darmstadt. Sequencing grade modified trypsin was from Promega, Madison, WI USA. Bicinchoninic acid assay (BCA) kit was acquired from Thermo Fisher Scientific, Dreieich, Germany. All chemicals for ultra-pure HPLC solvents such as formic acid (FA), trifluoroacetic acid (TFA) and acetonitrile (ACN) were obtained from Biosolve, Valkenswaard, The Netherlands.

Methods

Sample preparation and LC-MS/MS analysis

In total six samples (three TANGO2-patient derived fibroblasts and three control fibroblasts) were processed independently. Approximatively one million cells out of each sample were lysed in 200 µL of 50 mM Tris-HCl (pH 7.8) buffer containing 150 mM NaCl, 1 % SDS, and Complete Mini. Cell homogenization was carried by pipetting the mixture up and down until the complete solubilization of the pellet. In order to degrade the nucleic acids, benzonase (25 U/µL) together with 2mM MgCl2 was added to the lysate and incubated at 37 °C for 30 minutes. Next, samples were centrifuged at 4 °C for 30 minutes at 8000 g in order to separate cell debris from the protein extract. Protein concentration of the supernatant was determined by BCA assay according to the manufacturer’s protocol. Disulfide bonds were reduced by addition of 10 mM DTT at 56°C for 30 min, and free sulfhydryl bonds were alkylated with 30 mM IAA at room temperature (RT) in the dark for 30 min.

Sample preparation was performed using filter-aided sample preparation (FASP) as described previously with slight modifications (Roos et al, 2018). Briefly, cell lysate corresponding to 100 µg of protein was diluted 10-fold with freshly prepared 8 M urea/100 mM Tris-HCl (pH 8.5) buffer and placed on a Microcon centrifugal device (30 KDa cutoff). The device was centrifuged at 13,500 g at RT for 15 min. To eliminate residual SDS, three washing steps were carried out with 100 µL of 8 M urea/100 mM Tris-HCl (pH 8.5). To exchange the buffer, the filter was washed thrice with 100 µL of 50 mM NH4HCO3 (pH 7.8). Next, to the proteins 100 µL of proteolysis buffer: trypsin (Promega) (1:25 w/w, protease to substrate), 0.2 M GuHCl and 2 mM CaCl2 in 50 mM NH4HCO3 (pH 7.8), was added and samples were incubated at 37°C for 14 h. The generated tryptic peptides were recovered by centrifugation with 50 µL of 50 mM NH4HCO3 followed by 50 µL of ultra-pure water. The obtained tryptic peptides were acidified to a pH < 3.0 with 10% TFA and digests were quality controlled as described previously.

2 µg of each sample was analyzed using an Orbitrap Elite mass spectrometer coupled with an Ultimate 3000 nano RSLC system. Peptides were preconcentrated on a 100 µm x 2 cm C18 trapping column for 10 min using 0.1 % TFA (v/v) at a flow rate of 20 µL/min followed by separation on a 75 µm x 50 cm C18 main column (both Pepmap, Thermo Scientific) with: 1) a 130 min LC gradient ranging from 3-42 % of 84 % ACN, 0.1 % FA (v/v) at a flow rate of 230 nL/min.

All samples were measured in a data-dependent acquisition manner. In the OrbiElite MS survey scans were acquired in the Orbitrap from m/z 300 to 1500 at a resolution of 60,000 using the polysiloxane ion at m/z 371.101236 as lock mass3. The fifteen most intense signals were subjected to collision induced dissociation (CID) in the ion trap, taking into account a dynamic exclusion of 30s. CID spectra were acquired with normalized collision energy of 35 % and an activation time of 10 ms. AGC target values were set to 106 for MS1 and 104 for ion trap MS2 scans, and maximum injection times were set to 100 ms for both full MS and MS2 scans.

Label free data analysis

Data analysis of the acquired label free quantitative MS was performed using the Progenesis LC-MS software from Nonlinear Dynamics (Newcastle upon Tyne, U.K.). Raw files were imported and the alignment of the MS data was done automatically by the software by choosing one of the runs as a reference. After peak picking, only features within retention time and m/z windows from 0-130 and 300-1500 m/z, with charge states +2, +3, and +4 were considered for peptide statistics, analysis of variance (ANOVA) and principal component analysis (PCA). The MS/MS spectra were exported as peak lists and searched against a concatenated target/decoy version of the human Uniprot database, (downloaded on 22.07.2015, containing 20,273 target sequences) using Mascot 2.4 (Matrix Science), MS-GF+ Beta (v10282), and X!Tandem Jackhammer (2015.12.15.2) with the help of searchGUI 15.26.124. Trypsin with a maximum of two missed cleavages was selected as enzyme. Carbamidomethylation of Cys was set as fixed, acetylation of protein N-terminus, oxidation of Met and phosphorylation of Ser/Thr/Tyr were selected as variable modifications. MS and MS/MS tolerances were set to 10 ppm and 0.5 Da, respectively. Combined search results were filtered at a false discovery rate (FDR) of 1 % on the protein level and exported using the PeptideShaker software 1.16.17 (http://code.google.com/p/peptide-shaker/) features which allow a direct re-import of the quality-controlled data into Progenesis. Only proteins that were quantified with unique peptides were exported. Then, for each protein, the average of the normalized abundances (obtained from Progenesis) from was calculated to determine the ratios between the TANGO2-patient derived fibroblasts and control samples. Proteins identified with at least two unique peptides were classified as confident. Only proteins matching the following criteria were considered as regulated: which were (i) commonly quantified in all the replicates with (ii) an ANOVA p-value of <0.05 (Progenesis) and (iii) an average log2 ratio < 2.20 or > -2.55.

Data plotting and pathway analysis

All data was plotted using Origin 6.0 and Adobe Illustrator. Proteomaps were created based on a defined hierarchy tree from certain datasets using the online tool available (http://bionic-vis.biologie.uni-greifswald.de/). The annotation for this proteomaps are based on the KEGG database platform and each protein is shown by a polygon and functionally relevant proteins are arranged as neighbors. Additionally, polygon areas represent protein abundances weighted by protein size.

Supplementary Tables

Supplementary Table 1 Clinical hallmarks in reported individuals with bi-allelic *TANGO2* mutations

(Lalani et al. 2016; Kremer et al. 2016; Dines et al. 2018)

Key: n: number of reported cases; mt: month; y: year; TSH: thyroid stimulating hormone; ECG: electrocardiogram; MRI: magnetic resonance imaging.

| Number of reported cases | n= 38 |
| --- | --- |
| Sex | 22/38 female  16/38 male |
| Age of onset | 4 mt-8y |
| Developmental  Delay | yes: 36/38- 95%  not reported: 2/38 – 5% |
| Recurrent  metabolic crisis | yes: 27/38- 96%  not reported: 10/38- 26%  no: 1/38- 4% |
| Lactic  Acidosis | yes: 29/38- 76 %  not reported 7/38- 18%  normal 2/38- 6% |
| Blood glucose | hypoglycemia: 25/38- 66%  normoglycemia: 13/38- 34% |
| Acylcarnitine  Profile | unspecific elevation 11/38- 58%  not reported 22/38- 29%  normal 5/38- 13% |
| Respiratory  chain function  (Enzyme  activity) | normal 15/38- 63 %  reduced 3/38- 12%  not reported 20/38- 25% |
| Ammonia  Elevation | Elevated 16/38- 42 %  not reported 19/38- 50%;  normal 3/38- 8% |
| Transaminase  Elevation | 31/38- 82 %  not reported 7/38- 18% |
| Myopathy,  Rhabdomyolysis  (creatine kinase↑) | yes: 35/38- 92%  not reported: 3/38- 8 |
| TSH Elevation | 19/38- 50%  not reported 19/38- 50% |
| ECG changes/  Cardiac Arrhythmias  (Long QTc Interval/  Ventricular Tachycardia/  Torsade de Pointe) | yes: 24/38- 63 %  normal 4/38- 13%  not reported 9/24- 4% |
| Seizures | yes: 30/38- 78%  no: 8/38- 22% |
| Movement  Disorder  (Ataxia/  Extrapyramidal symptoms) | 27/38- 71%  not reported 11/29- 8% |
| Brain  abnormalities  (global brain atrophy MRI) | yes: 18/38- 47 %  no: 8/38- 11%  not reported: 12/38- 32% |
| Premature death | 11/38- 29 % |

Supplementary Table 2 Reported mutations in individuals with bi-allelic *TANGO2* mutations

*(Lalani et al. 2016; Kremer et al. 2016; Dines et al. 2018)*

Key: n: number of reported cases

| *Mutation variants in TANGO2* | *Number of individuals*  *(n=38), Ethnicity* |
| --- | --- |
| c.[460G>A];[460G>A]  p.[Gly154Arg] | 4 Hispanic |
| exons 3-9 del  (homozygous) | 5 European  12- ethnicity not reported |
| exons 3-9 del  (hemizygous) | 1, Caucasian (Subject 5 has a *de novo* 22q11.21 deletion) |
| exons 3-9 del;[460G>A]  p.[Gly154Arg] | 1, European/Hispanic |
| exons 3-9 del;c.[711-3C>G]  intron variant | 3, ethnicity not reported |
| exons 3-9 del; c.[265G>T]  p[G89C] | 1, ethnicity not reported |
| exons 3-9 del; 22q11.2 del | 1, ethnicity not reported |
| exons 3-9 del; exon 6 del | 1, ethnicity not reported |
| exons 4-6 del  (homozygous) | 2, Arab |
| c.[94C>T];[77G>A]  p.[R32*];[R26K] | 2, ethnicity not reported |
| c.[605+1G>A/wt];[605+1G>A/wt] | 1, Hispanic |
| c.[418C>T];[418C>T]  p.[Arg140*] | 1, Caucasian |
| c. [(56+1_57-1)_*1_?)del];  [(56+1_57-1)_*1_?)del]  p. [?];[?] | 1, European |
| c.[418delT];[(56+1_57-1)_*1_?)del]  p.[cys2Alafs*35];[?] | 1, European |
| c.[262C>T];[262C>T],  p.Arg88* *(novel)* | 1, Caucasian  (Subject 1) |
| c.[11-13delTCT p.(Phe5del)];  *(novel)* | 2, Hispanic  (Subject 2, 2.1) |
| c.[220A>C];[220A>C]  p.Thr74Pro *(novel)* | 1, Arab  (Subject 4) |
| Exon5/ Intron5;  c.[380+1G>A] *(novel)* | 2, Caucasian  (Subject 3, 3.1) |
| c.15_17del; p.Phe6del | 1, Afghani (Subject 6) |

***Novel:* mutations found in our subjects and not described previously**

Supplementary Table 3 Respiratory chain activity in skeletal muscle of the presented subjects (U/U CS: Unit/Unit Citrate Synthase; gram non-collagen protein: gNCP)

| *Respiratory chain activity*  *Related to citrate-synthase [quotient]* (normal range) | | | | |
| --- | --- | --- | --- | --- |
| OXPHOS Enzymes | *Subject 1* | *Subject 2.1* | *Subject 4* | *Subject 7* |
| Complex I  (U/U CS) | **0.13** (0.17-0.56) | **22** (107-560) | 47 (19-72) | 0.093 (0.104+0.036) |
| Complex II/III  (U/UCS) | **0.05** (0.08-0.45) | **19** (75-149) | 41 (30-76) | II: 0.126 (0.145+0.047)  III: 0.726 (0.554+0.345) |
| Complex IV  (U/U CS) | **0.68** (1.1-5.) | **240** (590-1300) | 5.23 (3.3-9.1) | 0.616 (1.124+0.511) |
| Citrate-synthase  (U/gNCP) | **123** (45-100) |  | 181 (85-179) |  |
| Coenzyme Q10  (nmol/U CS) | **1.2** (2.7-7.0) | **0.6** (2.5-5.3) | not determined | not determined |

Supplementary Table 4: overview on the regulated proteins and their proposed functions in TANGO2 deficient fibroblasts

| *Accession number* | *Protein* | *Peptide count* | *Unique peptides* | *p-Anova* | *Ratio: Pat./Cont.* | *Log2 ratio* | *Function* | *Disease associat.* |
| --- | --- | --- | --- | --- | --- | --- | --- | --- |
| O94907 | Dickkopf-related protein 1 (DKK1) | 2 | 2 | 0,0312 | 31,03 | 4,96 | Antagonizes Wnt signaling |  |
| Q15645 | Pachytene checkpoint protein 2 homolog (PCH2) | 2 | 2 | 0,0256 | 18,66 | 4,22 | Required for development of higher-order chromosome structures; needed for synaptonemal-complex formation | Mosaic variegated aneuploidy syndrome (MIM:617598) |
| Q8WUJ3 | Cell migration-inducing and hyaluronan-binding protein (CEMIP) | 6 | 5 | 0,0017 | 18,48 | 4,21 | Mediates depolymerization of hyaluronic acid via the cell membrane-associated clathrin-coated pit endocytic pathway; Interacts with HSPA5/BIP; the interaction induces calcium leakage from the endoplasmic reticulum and cell migration |  |
| P05106 | Integrin beta-3 (ITB3) | 4 | 4 | 0,0276 | 17,18 | 4,10 | Receptor for cytotactin, fibronectin, collagen, laminin etc | Glanzmann thrombasthenia (MIM:273800) |
| O00425 | Insulin-like growth factor 2 mRNA-binding protein 3 (IF2B3) | 3 | 2 | 0,0266 | 16,96 | 4,08 | RNA-binding factor that may recruit target transcripts to cytoplasmic protein-RNA complexes; binds to beta-actin transcripts |  |
| Q92621 | Nuclear pore complex protein Nup205 (NU205) | 1 | 1 | 0,0042 | 11,27 | 3,49 | Plays a role in the nuclear pore complex assembly and/or maintenance | Nephrotic syndrome (MIM:616893) |
| O94808 | Glutamine--fructose-6-phosphate aminotransferase 2 (GFPT2) | 6 | 2 | 0,0338 | 9,87 | 3,30 | Controls the flux of glucose into the hexosamine pathway |  |
| P08473 | Neprilysin (NEP_HUMAN) | 2 | 2 | 0,0075 | 7,25 | 2,86 | Thermolysin-like specificity, but is almost confined on acting on polypeptides of up to 30 amino acids | Charcot-Marie-Tooth disease 2T (MIM:617017) |
| Q13243 | Serine/arginine-rich splicing factor 5 (SRSF5) | 2 | 2 | 0,0166 | 7,13 | 2,83 | Plays a role in constitutive splicing and can modulate the selection of alternative splice sites |  |
| Q92626 | Peroxidasin homolog (PXDN) | 1 | 1 | 0,0293 | 6,30 | 2,65 | Displays low peroxidase activity and is likely to participate in H_2_O_2_ metabolism and peroxidative reactions in the cardiovascular system | Anterior segment dysgenesis (MIM:269400) |
| Q14320 | Protein FAM50A (FA50A) | 3 | 3 | 0,0421 | 5,83 | 2,54 | DNA-binding protein or transcriptional factor |  |
| Q9P2B4 | CTTNBP2 N-terminal-like protein (CT2NL) | 1 | 1 | 0,0038 | 5,61 | 2,49 | Colocalizes with stress fibers |  |
| Q15437 | Protein transport protein Sec23B (SC23B) | 1 | 1 | 0,0480 | 5,13 | 2,36 | Component of the coat protein complex II which promotes formation of transport vesicles from the ER | Cowden syndrome (MIM:616858) |
| Q13310 | Polyadenylate-binding protein 4 (PABP4_HUMAN) | 7 | 3 | 0,0001 | 4,92 | 2,30 | Packing of cytoplasmic mRNP granules containing untranslated mRNAs |  |
| P04818 | Thymidylate synthase (TYSY) | 2 | 2 | 0,0034 | 4,74 | 2,24 | Contributes to the *de novo* mitochondrial thymidylate biosynthesis pathway |  |
| P13995 | Bifunctional methylenetetrahydrofolate dehydrogenase/cyclohydrolase, mitochondrial (MTDC) | 1 | 1 | 0,0270 | 4,61 | 2,20 | Has very different kinetic properties than the larger NADP-dependent trifunctional enzyme and is unique in that it requires formation of an enzyme-magnesium complex to allow binding of NAD |  |
| P26006 | Integrin alpha-3 (ITA3) | 7 | 7 | 0,0051 | 0,17 | -2,55 | receptor for fibronectin, laminin, collagen, epiligrin, thrombospondin and CSPG4 | Interstitial lung disease, nephrotic syndrome, and epidermolysis bullosa (MIM:614748) |
| Q08357 | Sodium-dependent phosphate transporter 2 (S20A2) | 2 | 2 | 0,0437 | 0,17 | -2,57 | Phosphate transport by absorbing phosphate from interstitial fluid for cellular metabolism, signal transduction, and nucleic acid and lipid synthesis | Symmetric calcification in the basal ganglia and other brain regions associated with a wide spectrum of neuropsychiatric symptoms (MIM:213600) |
| Q06136 | 3-ketodihydrosphingosine reductase (KDSR) | 1 | 1 | 0,0121 | 0,16 | -2,61 | Sphingolipid metabolism | Erythrokeratodermia variabilis et progressiva (MIM:617526) |
| Q7Z3B1 | Neuronal growth regulator 1 (NEGR1) | 1 | 1 | 0,0431 | 0,16 | -2,64 | Trans-neural growth-promoting factor in regenerative axon sprouting in the mammalian brain |  |
| O43772 | Mitochondrial carnitine/acylcarnitine carrier protein (MCAT) | 1 | 1 | 0,0077 | 0,15 | -2,74 |  | Carnitine-acylcarnitine translocase deficiency associated with neurologic abnormalities, cardiomyopathy, arrhythmias, skeletal muscle damage, liver dysfunction and episodes of life-threatening coma (MIM:212138) |
| Q9P121 | Neurotrimin (NTRI) | 2 | 2 | 0,0234 | 0,14 | -2,80 | Neural cell adhesion molecule |  |
| Q13232 | Nucleoside diphosphate kinase 3 (NDK3) | 1 | 1 | 0,0189 | 0,13 | -2,94 | Synthesis of nucleoside triphosphates other than ATP |  |
| Q16647 | Prostacyclin synthase (PTGIS) | 8 | 8 | 0,0316 | 0,13 | -2,95 | Catalyzes isomerization of prostaglandin to prostacyclin | Essential hypertension (MIM:145500) |
| P13591 | Neural cell adhesion molecule 1 (NCAM1) | 3 | 3 | 0,0025 | 0,13 | -2,97 | Cell adhesion molecule involved in neuron-neuron adhesion, neurite fasciculation, outgrowth of neurites |  |
| P04156 | Major prion protein (PRIO) | 1 | 1 | 0,0169 | 0,13 | -2,99 | Neuronal development and synaptic plasticity; May be required for neuronal myelin sheath maintenance | Creutzfeldt-Jakob disease (MIM:123400) |
| Q9UBP4 | Dickkopf-related protein 3 (DKK3) | 2 | 2 | 0,0001 | 0,12 | -3,00 | Antagonizes canonical Wnt |  |
| Q8N2F6 | Armadillo repeat-containing protein 10 (ARM10) | 1 | 1 | 0,0032 | 0,12 | -3,05 | May play a role in cell survival and cell growth |  |
| Q02083 | N-acylethanolamine-hydrolyzing acid amidase (NAAA) | 1 | 1 | 0,0033 | 0,12 | -3,06 | Degrades bioactive fatty acid amides to their corresponding acids |  |
| Q9UMS6 | Synaptopodin-2 (SYNP2) | 7 | 7 | 0,0336 | 0,12 | -3,07 | Induces formation of F-actin networks in an isoform-specific manner |  |
| Q14728 | Major facilitator superfamily domain-containing protein 10 (MFS10) | 1 | 1 | 0,001597 | 0,12 | -3,11 | Promotes muscle cell proliferation and de-differentiation |  |
| Q16527 | Cysteine and glycine-rich protein 2 (CSRP2) | 3 | 3 | 0,0330 | 0,12 | -3,17 |  |  |
| Q02252 | Methylmalonate-semialdehyde dehydrogenase [acylating], mitochondrial (MMSA) | 1 | 1 | 0,0432 | 0,11 | -3,25 | Plays a role in valine and pyrimidine metabolism. Binds fatty acyl-CoA | Methylmalonate semialdehyde dehydrogenase deficiency (MIM:614105) |
| P17302 | Gap junction alpha-1 protein (CXA1) | 4 | 3 | 0,0013 | 0,10 | -3,28 | Localizes at the intercalated disk in cardiomyocytes; acts in cardioprotection | Oculodentodigital dysplasia (MIM:164200) |
| O95810 | Caveolae-associated protein 2( CAVN2) | 8 | 8 | 0,0159 | 0,10 | -3,28 | Plays an important role in caveolar biogenesis and morphology |  |
| O75056 | Syndecan-3 (SDC3) | 1 | 1 | 0,0068 | 0,10 | -3,33 | organization of cell shape by affecting the actin cytoskeleton |  |
| P30038 | Delta-1-pyrroline-5-carboxylate dehydrogenase, mitochondrial (AL4A1) | 6 | 6 | 0,0152 | 0,10 | -3,33 | Irreversible conversion of delta-1-pyrroline-5-carboxylate to glutamate | Hyperprolinemia 2 (HYRPRO2): affected individuals can exhibit neurological manifestations (MIM:239510) |
| Q9Y5S1 | Transient receptor potential cation channel subfamily V member 2 (TRPV2) | 4 | 4 | 0,0411 | 0,10 | -3,37 | Calcium-permeable, non-selective cation channel with an outward rectification |  |
| P24385 | G1/S-specific cyclin-D1 (CCND1) | 3 | 3 | 0,0036 | 0,09 | -3,44 | Regulatory component of the cyclin D1-CDK4 complex that phosphorylates and inhibits members of the retinoblastoma protein family | Multiple myeloma (MIM:254500) |
| P56199 | Integrin alpha-1 (ITA1) | 4 | 3 | 0,0009 | 0,09 | -3,55 | Integrin alpha-1/beta-1 is a receptor for laminin and collagen |  |
| Q7Z4F1 | Low-density lipoprotein receptor-related protein 10 (LRP10) | 1 | 1 | 0,0038 | 0,08 | -3,61 | Probable receptor, which is involved in the internalization of lipophilic molecules and/or signal transduction |  |
| P51648 | Fatty aldehyde dehydrogenase (AL3A2) | 1 | 1 | 0,0034 | 0,08 | -3,62 | Catalyzes the oxidation of long-chain aliphatic aldehydes to fatty acids | Sjoegren-Larsson syndrome (MIM:270200) |
| P24593 | Insulin-like growth factor-binding protein 5 (IBP5) | 1 | 1 | 0,0441 | 0,08 | -3,62 | IGF-binding proteins prolong the half-life of the IGFs and have been shown to either inhibit or stimulate the growth promoting effects |  |
| P29317 | Ephrin type-A receptor 2 (EPHA2) | 3 | 3 | 0,0101 | 0,08 | -3,69 | Receptor tyrosine kinase which binds promiscuously membrane-bound ephrin-A family ligands residing on adjacent cells, leading to contact-dependent bidirectional signaling into neighboring cells | Cataract (MIM:116600) |
| Q15746 | Myosin light chain kinase, smooth muscle (MYLK) | 12 | 11 | 0,0269 | 0,08 | -3,73 | Calcium/calmodulin-dependent myosin light chain kinase implicated in (smooth) muscle contraction via phosphorylation of myosin light chains; Also regulates actin-myosin interaction through a non-kinase activity | Familial thoracic aortic aneurysm (MIM:613780) |
| Q92743 | Serine protease HTRA1 (HTRA1) | 8 | 6 | 0,0113 | 0,07 | -3,80 | Serine protease with a variety of targets, including extracellular matrix proteins such as fibronectin | CARASIL (MIM:600142) & age-related macular degeneration (MIM:610149) |
| Q6UX15 | Layilin (LAYN) | 1 | 1 | 0,0027 | 0,07 | -3,90 | Receptor for hyaluronate |  |
| P59768 | Guanine nucleotide-binding protein G(I)/G(S)/G(O) subunit gamma-2 (GBG2) | 1 | 1 | 0,0072 | 0,06 | -4,13 | Modulator or transducer in various transmembrane signaling systems. |  |
| Q7Z5R6 | Amyloid beta A4 precursor protein-binding family B member 1-interacting protein (AB1IP) | 1 | 1 | 0,0021 | 0,06 | -4,13 | Signal transduction from Ras activation to actin cytoskeletal remodeling |  |
| Q13938 | Calcyphosin (CAYP1) | 1 | 1 | 0,0368 | 0,05 | -4,27 | Calcium-binding protein; may play a role in cellular signaling events |  |
| P27658 | Collagen alpha-1(VIII) chain (CO8A1) | 3 | 3 | 0,0198 | 0,04 | -4,56 | Necessary for migration and proliferation of vascular smooth muscle cells |  |
| Q15147 | 1-phosphatidylinositol 4,5-bisphosphate phosphodiesterase beta-4 (PLCB4) | 2 | 2 | 0,0005 | 0,04 | -4,79 | Production of the second messenger molecules diacylglycerol (DAG) and inositol 1,4,5-trisphosphate (IP3) | Auriculocondylar syndrome (MIM:614669) |
| Q9NRR1 | Cytokine-like protein 1 (CYTL1) | 1 | 1 | 0,0361 | 0,02 | -6,06 | Involved in chondrogenesis and cartilage development |  |
| Q8ND94 | LRRN4 C-terminal-like protein (LRN4L) | 2 | 2 | 0,0338 | 0,01 | -6,66 |  |  |
| Q9BY67 | Cell adhesion molecule 1 (CADM1) | 1 | 1 | 0,0010 | 0,00 | -14,04 | Mediates homophilic cell-cell adhesion in a Ca2+-independent manner. |  |

References

Bartsakoulia, M., Pyle, A., Troncoso-Chandia et al (2018) A novel mechanism causing imbalance of mitochondrial fusion and fission in human myopathies. *Human Molecular Genetics*, **27**, 1186-1195.

Roos, A., Thompson, R., Horvath, R., Lochmuller, H. & Sickmann, A. (2018) Intersection of Proteomics and Genomics to "Solve the Unsolved" in Rare Disorders such as Neurodegenerative and Neuromuscular Diseases. *Proteomics Clin Appl*, **12**.
